# Injectable hydrogel with doxorubicin-loaded ZIF-8 nanoparticles for tumor postoperative treatments and wound repair

**DOI:** 10.1038/s41598-024-57664-0

**Published:** 2024-05-01

**Authors:** Qiang Zhang, Yu Zhang, Hui Chen, Lei-Na Sun, Bin Zhang, Dong-Sheng Yue, Chang-Li Wang, Zhen-Fa Zhang

**Affiliations:** 1 Department of Lung Cancer, Tianjin Medical University Cancer Institute and Hospital, National Clinical Research Center for Cancer, Tianjin, China; 2 Key Laboratory of Cancer Prevention and Therapy, Tianjin, China; 3 Tianjin Lung Cancer Center, Tianjin, China; 4 Tianjin’s Clinical Research Center for Cancer, Tianjin, China; 5 Department of Pathology, Tianjin Medical University Cancer Institute & Hospital, Tianjin, China

**Keywords:** Injectable hydrogel, ROS scavenger, Tumor recurrence, Wound repair, Nanoparticle, Lung cancer, Drug discovery, Medical research, Oncology

## Abstract

The need for tumor postoperative treatments aimed at recurrence prevention and tissue regeneration have raised wide considerations in the context of the design and functionalization of implants. Herein, an injectable hydrogel system encapsulated with anti-tumor, anti-oxidant dual functional nanoparticles has been developed in order to prevent tumor relapse after surgery and promote wound repair. The utilization of biocompatible gelatin methacryloyl (GelMA) was geared towards localized therapeutic intervention. Zeolitic imidazolate framework-8@ceric oxide (ZIF-8@CeO2, ZC) nanoparticles (NPs) were purposefully devised for their proficiency as reactive oxygen species (ROS) scavengers. Furthermore, injectable GelMA hydrogels loaded with ZC NPs carrying doxorubicin (ZC-DOX@GEL) were tailored as multifunctional postoperative implants, ensuring the efficacious eradication of residual tumor cells and alleviation of oxidative stress. In vitro and in vivo experiments were conducted to substantiate the efficacy in cancer cell elimination and the prevention of tumor recurrence through the synergistic chemotherapy approach employed with ZC-DOX@GEL. The acceleration of tissue regeneration and in vitro ROS scavenging attributes of ZC@GEL were corroborated using rat models of wound healing. The results underscore the potential of the multifaceted hydrogels presented herein for their promising application in tumor postoperative treatments.

## Introduction

Surgical resection stands as the prevailing modality for the treatment of diverse solid tumors. However, patients are confronted with the inherent risk of recurrent tumors and postoperative wounds^1,2^. Tumor recurrence, primarily attributed to the presence of residual tumor cells post-surgery, entails significant mortality implications^3^. Conventional chemotherapy is frequently employed to target residual tumor cells; nonetheless, its efficacy is constrained by suboptimal delivery efficiency and non-selective cytotoxicity^4^. Moreover, chronic wounds at surgical sites may be concomitant with sustained inflammation, potentially exacerbating the likelihood of tumor recurrence^3,5,6^. Hence, there is an exigency to devise a postoperative adjuvant therapy platform proficient in the efficient eradication of residual tumor cells while concurrently establishing an optimal microenvironment at tumor resection sites to expedite wound healing.

In the realm of advanced postoperative tumor treatment, a paramount objective is to attain a heightened drug concentration precisely at the target tumor resection site while mitigating systemic toxicity. Among the diverse materials employed in the formulation of nanomedicines, metal–organic frameworks (MOFs) have emerged as a pivotal category of crystalline porous materials. These frameworks are constructed through the coordination of metal ions with organic linkers and have garnered substantial attention as apt nanocarriers for drug delivery over the past decade^7–9^. Zeolitic imidazolate framework-8 (ZIF-8), a subclass of MOFs comprising Zn ions and 2-MeIM, has been extensively studied in drug delivery owing to its remarkable attributes, including high biocompatibility, pH-responsive biodegradability, and the capability for endo/lysosomal escape^10^. Notably, ZIF-8 exhibits stability in a neutral pH environment (pH 7.4), but undergoes degradation in tumor tissues characterized by a mildly acidic pH range of 5.5–6.0^11^. This property enables rapid degradation into Zn ions and 2-MeIM, thereby facilitating the release of encapsulated drug substances ^27,28^. Consequently, ZIF-8 holds immense promise for cancer treatment and has found application in this domain^12^. Concurrently, ceric oxide, a widely employed nano-enzyme, showcases exceptional anti-oxidant capabilities akin to superoxide dismutase (SOD) and catalase (CAT) activities, primarily attributed to the facile switching between surface Ce (III) and Ce (IV) valency. This endows it with the ability to scavenge hydroxyl radicals and nitric oxide radicals^13,14^, thereby manifesting a tissue repair effect associated with ROS scavenging in numerous biologically relevant contexts.

On the other side, many aggressive therapeutic modalities, such as black phosphorus, glucose oxidase, photosensitizers, radiosensitizers, and pharmaceuticals, have been incorporated into hydrogel matrices to facilitate combinational therapies against residual tumors. However, this approach often induces pronounced local stress responses, including chronic inflammation, which is detrimental to postoperative wound healing^15–17^. Hence, integrating chemotherapy with anti-inflammatory therapy represents an alternative strategy for postoperative tumor treatments, albeit necessitating further preclinical validation. Recent advancements have led to the development of multifunctional platforms that amalgamate diverse therapeutic modalities with anti-inflammatory interventions, aimed at preventing tumor recurrence while expediting wound healing. For instance, hydrogels containing doxorubicin@prussian blue nanoparticles have been reported to confer both chemo-photothermal therapy and anti-inflammatory effects^18^. To address the limitation of short-term drug release associated with conventional agents in resolving comparatively prolonged inflammation, artificial enzymes with ROS-scavenging capabilities present themselves as promising alternatives. These entities offer enduring and efficacious anti-inflammatory effects alongside catalytic activity, concurrently sidestepping potential side effects^19^. Injectable hydrogels, as biomaterials, have recently demonstrated remarkable potential across diverse applications including drug delivery, wound dressings, and tissue fillers. Their high moisture content, optimal gas exchange properties, and capacity to emulate natural extracellular matrix (ECM) structures while responding to external stimuli underscore their appeal^20,21^. Consequently, hydrogels with shear-thinning attributes can proficiently stabilize cargo such as nanoparticles and cells in a solid-state state, allowing for convenient injection without the need for on-site preparation^22^. Synthetically integrating hydrogels with nanoparticles to create a hybrid biomimetic system further capitalizes on the synergistic advantages of both components^23^.

**Figure 1:**
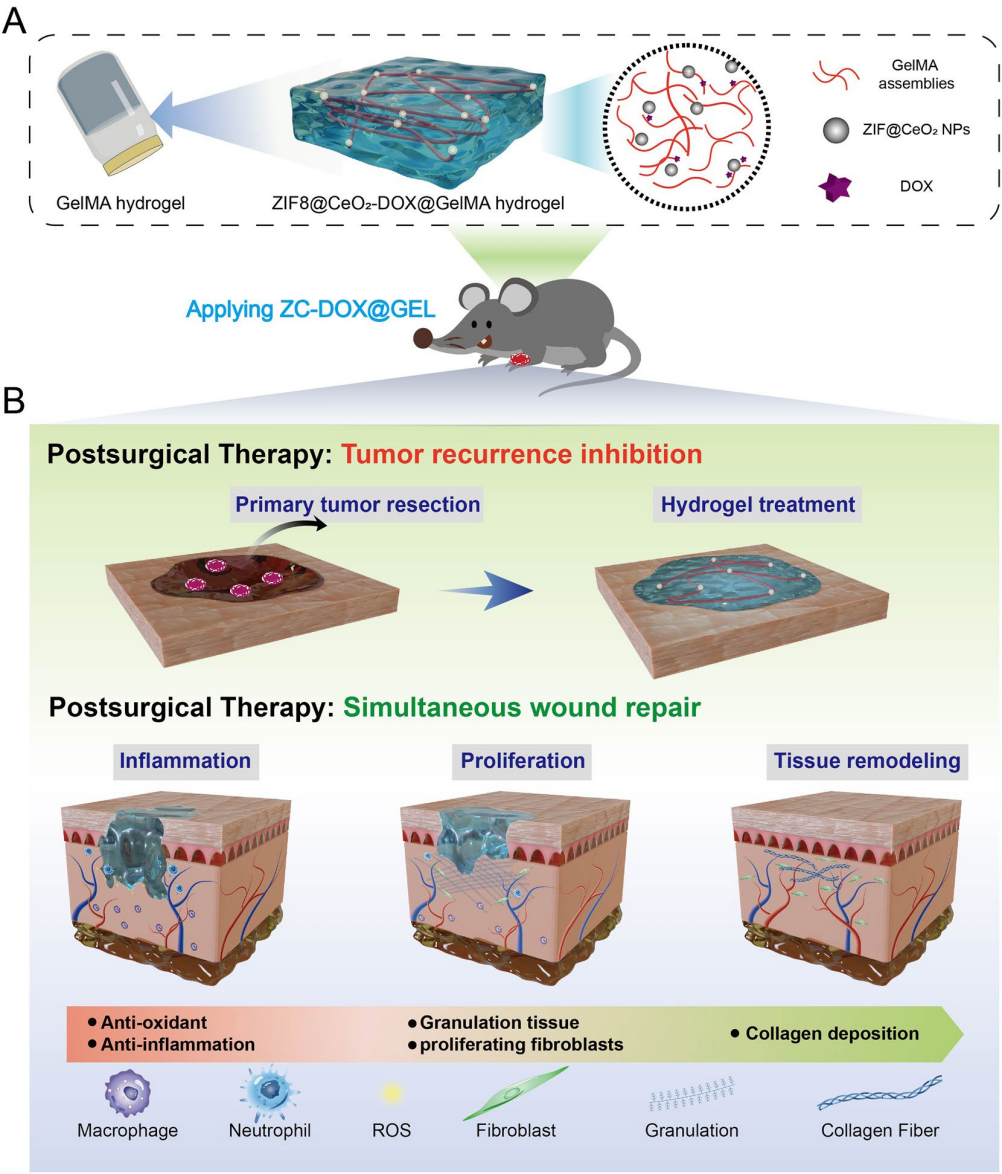
Schematic illustration of (**A**) GelMA hydrogel loaded with DOX@ZIF8@CeO_2_ NPs and (**B**) Postoperative treatment: preventing tumor recurrence and promoting wound healing.

This study endeavors to fabricate an injectable inflammation-responsive hydrogel, specifically, a DOX-loaded zeolitic imidazolate framework-8@ceric oxide nanoparticle (ZIF-8@CeO_2_, ZC) embedded within a GelMA hydrogel (ZC-DOX@GEL), designed for tumor treatment (Fig. 1). ZC-DOX@GEL, comprising biocompatible constituents, will exhibit attributes of biocompatibility, facile administration (including ease of preparation and temperature modulation), and potent efficacy in tumor therapy. Furthermore, the generated ZC-DOX nanoparticle is anticipated to augment the therapeutic effectiveness of DOX. Concurrently, the sustained presence of hydrogels at tumor sites is poised to prolong drug release and modulate the tumor microenvironment, culminating in enhanced anti-tumor efficacy. Additionally, ameliorating oxidative stress and bolstering the accumulation and proliferation of fibroblasts at wound sites bodes well for the substantial advancement of wound healing. This endeavor has the potential to yield a multifaceted inflammation-responsive biomaterial platform with applications in postoperative adjuvant therapy and wound healing.

## Materials and methods

### Materials

All material details can be found in supplementary information. All experimental protocols were approved by the Animal Use and Care Committee of Tianjin Tumor Hospital. All methods were carried out in accordance with relevant guidelines and regulations.

### Synthesis of GelMA hydrogel

A detailed procedure for the synthesis of GelMA is available in supplementary information.

### Synthesis of ZIF-8@CeO_2_ and DOX@ ZIF-8@CeO_2_ nanoparticles (NPs)

Ceric oxide nanoparticles (CeO_2_ NPs) were synthesized following established protocols with slight modifications^24^. Specifically, 430 mg of cerium (III) acetate hydrate and 3.2 g of oleylamine were dissolved in 15 mL of xylenes and vigorously stirred for 24 h. The mixture was then gradually heated to 90 °C, and 1 mL of deionized (DI) water was swiftly injected. The solution was maintained at 90 °C for 3 h. Ethanol was employed to precipitate the CeO_2_ NPs, which were subsequently collected through centrifugation.

The synthesis of DOX@ZIF-8@ CeO_2_ (ZC-DOX) followed a previously reported procedure with some adaptations^25^. Specifically, 500 mg of 2-methylimidazole and 5 mg of doxorubicin were combined in 4 mL of methanol and stirred for 10 min at room temperature. Subsequently, 1 mL of a water solution containing 2.5 mg of Zn(NO_3_)_2_·6H_2_O was introduced to the mixture. The reaction was vigorously stirred for 15 min, resulting in an emulsion-like suspension. The nano-cerium oxide particles and polyvinylpyrrolidone were dispersed in methanol and added to the emulsion-like suspension, followed by stirring for an additional 5 min at room temperature. The final product was obtained through centrifugation, and impurities were removed by washing with methanol. ZIF-8@CeO_2_ (ZC) was synthesized using the same protocol.

### Structural characterization and properties tests

Scanning electron microscopy (SEM) was utilized for the observation of morphological details of ZC-DOX NPs and the intersection network and elemental mapping of the hydrogel. The structures of the ZC-DOX NPs were characterized using transmission electron microscopy (TEM, JEM2100, JEOL, Japan).

The particle sizes and zeta potentials of ZIF-8@CeO_2_ and DOX@ZIF-8@CeO_2_ NPs were determined via dynamic light scattering (DLS) at 25℃, utilizing a Zetasizer Nano ZS (Malvern, UK) equipped with a 633 nm laser and a detector set at a detection angle of 173°. Each sample was measured in triplicate.

To assess drug-release kinetics, 3 mL of ZC-DOX@GEL was immersed in 20 mL of PBS containing 1 mg/mL Tween 80, maintained at 37℃ within a tube. Subsequently, 3 mL of the solution was analyzed using UV–vis spectroscopy to quantify the released drug amount, after which it was returned to the tube.

The infrared spectrum of the hydrogels was acquired by combining freeze-dried gel powders with KBr, using a Fourier transform spectrometer (FTIR, Bruck EQUINOX55) with a measurement wavelength range spanning from 4000 to 400 cm^−1^.

The rheological properties of ZC-DOX@GEL hydrogel were investigated utilizing a TA rheometer (AR G2, USA). Typically, the hydrogel was positioned on a parallel-plate with a 20-mm diameter, and the modulus G’ and G’’ were recorded at 37 °C while varying the scanning frequency from 0.1 to 10 Hz, with three measurements taken per group.

### In vitro cell experiments

Cy5-labeled DOX was employed to monitor cellular uptake using a fluorescence microscope. 4T1 cells were seeded into 6-well plates at a density of 5 × 10^5^ cells per well, incubated overnight, and then treated with Cy5-DOX@ZIF-8@ CeO_2_ for 4 h. Subsequently, cells were washed and stained with 1 M DAPI for 30 min, followed by imaging with a fluorescence microscope (Nikon, Japan).

L929 fibroblasts and human epithelial cells (MCF-10A) were seeded into 96-well plates (1 × 10^4^ cells per well) pretreated with various hydrogel groups and cultured for 1, 3, and 7 days, respectively. Subsequently, a CCK-8 assay was conducted on the cells, and absorbance at 450 nm was measured using a microplate reader.

To assess tumor cell cytotoxicity, 2 × 10^4^ 4T1 cells were seeded in 48-well plates with 1 mL complete DMEM medium. After 12 h of incubation, 50 μL of various hydrogels or PBS were added to the wells. After an additional 24 h of incubation, experimental groups were subjected to live/dead staining using Calcein-AM/PI.

Transwell chamber assays were performed using Transwell permeable plates (Corning, USA) consisting of poly- carbonate Transwell inserts (8 μm pore diameter) and a 24-well plate. The 4T1 cells (2 × 10^4^) were seeded in the upper half of the insert membrane, then we implanted various hydrogel in the lower compartment of the 24-well plate and cultured for 24 h. The cells were fixed with 4% paraformaldehyde for 30 min. After the cells were rinsed with PBS, 0.1% crystal violet staining solution was used to stain them for 10 min. Images of migrating cells in the lower half of each insert were observed and captured, and the number of cells that migrated to the lower part of the insert was counted in three microscopic views per well. Subsequently, the difference in the mean cell number per well was analyzed.

For the evaluation of ROS scavenging ability and the resulting cell-protective effects, 1 × 10^5^ BMDMs cells were seeded in 48-well plates with 1 mL complete DMEM medium. After 12 h of incubation, the medium was replaced with 1 mL complete DMEM medium containing 1 mM H_2_O_2_, along with 100 μL of various hydrogels or PBS. After 2 h of incubation, the hydrogels were removed, and the plates were gently washed with PBS. The cells were stained with DCFH-DA and Hoechst 33342, then observed under a fluorescence microscope.

For the quantitative analysis of the anti-inflammatory effect of these hydrogels, RT-PCR was employed. RAW264.7 cells were seeded at a density of 2 × 10^5^ cells/well in 6-well plates and cultured with or without hydrogels overnight. Subsequently, they were treated with or without 0.5 μg/mL LPS for 24 h. After stimulation, we extracted total RNA by a Cell Total RNA Isolation Kit (Foregene, China), and converted total RNA to complementary DNA using 5 × RT Master Mix (Toyobo, Osaka, Japan). RT-PCR was performed on a StepOneTM Real-Time PCR system (Applied Biosystems, USA) using 2 × RealStar Green Fast mixture (Genstar, Beijing, China) according to the standard procedure. The primer sequences for IL-6, iNOS, Arg-1, and CD206 are shown in Table S1. To detect the reliability of the primers, we established fusion curves for each reaction system, and there was no nonspecific amplification in the dissolution curves. Relative mRNA expression was normalized to that of the housekeeping gene GAPDH and calculated by using the 2- ΔΔCT method.

### Tumor resection and postoperative prevention

To investigate the tumor therapeutic effect of hydrogels, incomplete resection of a breast cancer model was successfully established according to a study of Tong et al.^18^ with some modification. In detail, orthotopic breast cancer-bearing Balb/c mice (18–25 g, 4–6 weeks old) were established by injecting 5 × 10^5^ 4T1 cells into the mammary fat pad under the right breast. Tumors were surgically removed after reaching a volume of 100 mm^3^ under anesthesia (leaving about 5% of the tumor mass). On the second day post-surgery, 5 × 10^5^ 4T1 cells incubated in 50 μL of matrigel were injected into the vicinity of the surgical sites. Subsequently, 100 μL of various hydrogels or PBS were administered to the surgical sites. For DOX@GEL group, every mouse was treated with 100 μL GelMA hydrogels containing 28.2 μg DOX. For ZC-DOX@GEL group, every mouse was treated with 100 μL ZC-DOX@GEL hydrogels containing 100 μg ZC and 28.2 μg DOX. The mice were categorized into four groups based on different materials and phototherapeutic interventions: PBS, GEL, DOX@GEL, and ZC-DOX@GEL. Body weights and tumor volumes were assessed every 3 days. Tumor size was calculated according to the following formula:

$${\text{Tumor}}\,{\text{volume}}\left( {{\text{mm}}^{{\text{3}}} } \right) = {\text{width}}^{{\text{2}}} \times {\text{length}}/{\text{2}}.$$

On the 21st day following postoperative treatments, the mice were humanely euthanized by neck dislocation, and their heart, liver, spleen, lung, kidney tissues were excised for further analysis.

### Wound healing assays

A full-thickness excisional skin model was established in male SD rats (6–8 weeks old) to evaluate the wound healing potential of the hydrogels. Briefly, the rats were anesthetized, shaved, and cutaneous wounds were created using an 8 mm diameter biopsy punch. Subsequently, the rats were randomly assigned to one of three treatment groups (n = 3): PBS treatment alone, GelMA, and ZC@GelMA. At designated time points, skin samples from the wound area were collected and embedded in paraffin. The histopathological analyses of the samples were conducted using H&E and MassFon staining.

### Statistical analysis

All the data values were expressed as the mean ± standard deviation (SD). The inter-group differences in the experiments were evaluated using a two-tailed t-test through GraphPad Prism 6 software. Statistical significance was indicated as *p < 0.05, **p < 0.01, ***p < 0.001, and ****p < 0.0001, respectively.

### Ethics approval and consent to participate

All experimental protocols were approved by the Animal Use and Care Committee of Tianjin Tumor Hospital.

### Consent for publication

All authors have reviewed the final version of the manuscript and approved it for publication. The study is reported in accordance with ARRIVE guidelines.

## Results

### Preparation and characterization of ZC-DOX@GEL

**Figure 2:**
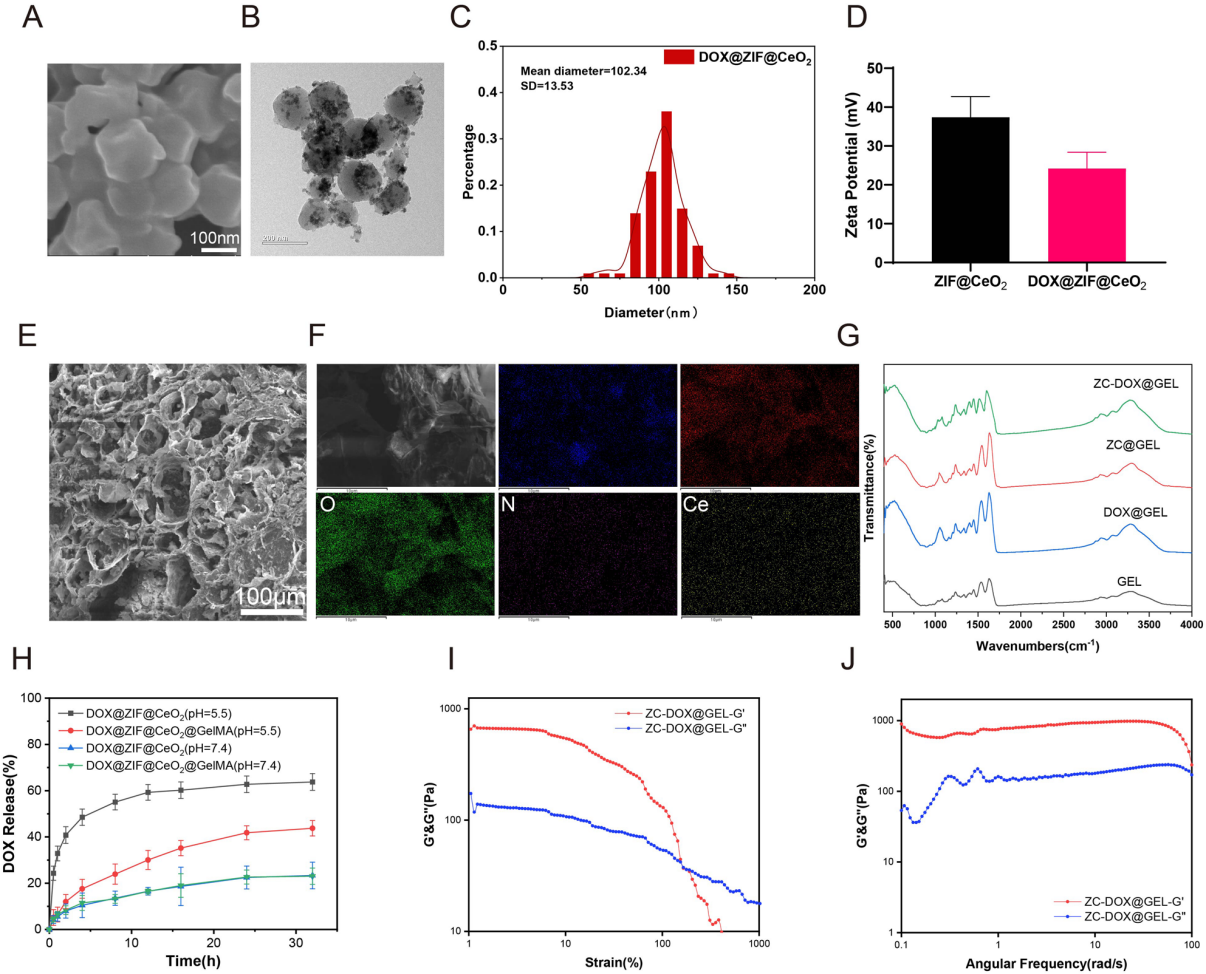
Characterization of DOX@ZIF8@CeO_2_@GelMA (ZC-DOX@GEL). (**A**) SEM image of ZC-DOX. Scale bar, 100 nm. (**B**) TEM image of ZC-DOX. Scale bar, 200 nm. (**C**) Particle size distribution of DOX@ZIF8@CeO_2_ NPs. (**D**) Zeta potential of ZIF8@CeO_2_ and DOX@ZIF8@CeO_2_ NPs. (**E**) SEM image of lyophilized cross-section and (**F**) elemental mapping of ZC-DOX@GEL. Scale bar, 100 μm. (**G**) Fourier transform infrared (FTIR) spectra of GEL(GelMA), DOX@GEL, ZC@GEL, and ZC-DOX@GEL hydrogels. (**H**) Cumulative DOX release profiles of DOX@ZIF8@CeO_2_ and ZC-DOX@GEL at different pH conditions. Rheological properties of ZC-DOX@GEL hydrogels, (**I**) The strain sweep test of hydrogel; (**J**) The frequency sweep test of hydrogel.

The ZC-DOX NPs were synthesized using the previously reported method^25^. They exhibited a spherical structure with a size of approximately 102 nm, as observed through scanning electron microscopy (SEM) (Fig. 2A and C). The morphology of ZC-DOX NPs was also observed by transmission electron microscopy (TEM) (Fig. 2B), exhibiting a regular circular shape with uniform dispersed CeO_2_ particles attached to its core structure surface. The zeta potential of DOX@ZIF-8@CeO_2_ NPs (37.4 ± 4.7 mV) was slightly higher than that of ZIF-8@CeO_2_ NPs (24.2 ± 3.7 mV), indicating the successful encapsulation of the DOX drugs (Fig. 2D). The elemental distribution in ZC-DOX@GEL was investigated using SEM (Fig. 2E) and energy dispersive spectrometry (EDS) mapping. After incorporating DOX@ZIF-8@CeO_2_ MOFs, the results indicated the presence of common elements such as C, Zn, and N inherited from ZIF-8, and Ce exclusively originating from CeO_2_, as shown in the EDS mapping (Fig. 2F). In the Fourier transform infrared spectrometer (FTIR) data, the bands at 995, 1311, and 1579 cm^−1^ corresponded to the C-H stretching of 2-MeIM, and the band at 420 cm^−1^ was attributed to the Zn-N stretch (Fig. 2G).

ZIF-8 NPs are pH-responsive due to the dissociation of the coordination between Zn^2+^ and 2-MeIM in acidic environments^26^. This property can be utilized as a targeting mechanism, where the release of the loaded drug from ZIF-8 NPs is triggered at tumor sites owing to their relatively lower pH (acidic environment) compared to normal tissues. Furthermore, we conducted another study to determine the effect of the nanoparticles on the loading capacity of the DOX. The drug loading rate and the encapsulation rate of the ZIF8@CeO2 nanoparticles and the DOX with different mass ratios (m (NPs): m (DOX)) were explored and summarized as shown in Table (shown below), which showed that the ZIF8@CeO2 nanoparticles exhibited excellent encapsulation efficiency for the DOX (> 63.9%). When the mass ratio of m (NPs): m (DOX) was 1:0.5, the final DOX drug load efficiency reached up to 28.2% (Table 1). This proves that the encapsulation of nanoparticles is effective.

**Table 1:** Encapsulation rate and drug loading rate analysis of nanoparticles.

ZIF8@CeO_2_: DOX	Encapsulation efficiency (%)	Drug loading efficiency (%)
1:0.1	76.5	12.7
1:0.2	64.2	21.4
1:0.5	63.9	28.2

Data were displayed as mean ± SD (n = 3). SD = 0.05.

To investigate the pH-responsive behavior of ZIF-8 NPs, an in vitro release study of DOX was conducted in PBS at various pH values (pH 5.5 and pH 7.4). The release rate of DOX from DOX@ZIF-8@CeO_2_ NPs in pH 7.4 PBS was only 23% of the total amount in the NPs after 32 h of incubation. However, the cumulative drug release was 63% in pH 5.5 PBS, indicating a significantly increased release rate under acidic conditions (Fig. 2H). Meanwhile, the NPs incorporated in the hydrogel demonstrated a prolonged release profile, which was beneficial for maintaining local drug concentrations at the tumor treatment site. Dynamic rheological curves (Fig. 2I,J) of the ZC-DOX@GEL sample demonstrated that G’ (storage modulus) was greater than G" (loss or viscous modulus), indicating the formation of a stable hydrogel structure.

### In vitro anticancer effects and cytocompatibility of ZC-DOX@GEL

**Figure 3:**
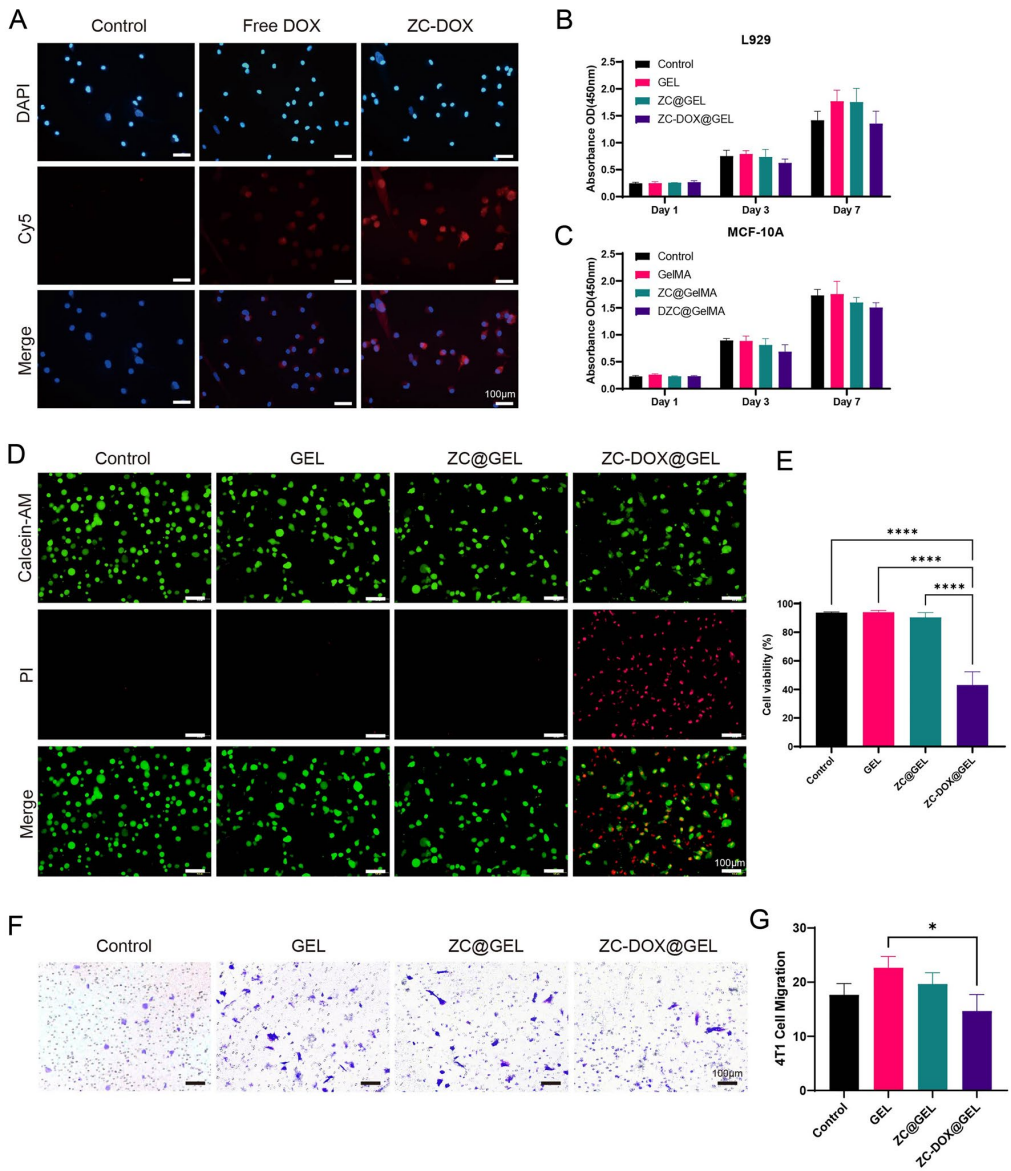
In vitro anticancer activity tests. (**A**) CLSM images of cellular uptake of DOX and DOX@ZIF8@CeO_2_. (**B**) Cell viability of L929 with different treatments. (**C**) Cell viability of MCF-10A with different treatments. (**D**) Live/dead 4T1 cell viability evaluation of Control, GEL, ZC@GEL and ZC-DOX@GEL. (**E**) Quantitatively analysis of 4T1 cell viability. (**F**) Crystal violet staining of the migrated 4T1 cell. (**G**) Quantitatively analysis of migrated 4T1 cell in different groups (n = 3).

The cellular uptake of ZC-DOX NPs was assessed via fluorescence microscopy in 4T1 cells. Cy5-labelled DOX (red) was encapsulated within ZIF-8 NPs instead of DOX for fluorescence detection, and the cell nuclei were stained with DAPI (blue). As depicted in Fig. 3A, the red fluorescence intensity of Cy5-DOX and Cy5-labelled ZC-DOX were notably higher than that in the Control group. Furthermore, the Cy5-DOX@ZIF-8@CeO_2_ NPs group exhibited a more robust fluorescent intensity compared to the Cy5-DOX group, indicating that Cy5-DOX@ZIF-8@CeO_2_ NPs enhance cellular internalization.

Good biocompatibility is a critical factor for materials used in biomedical applications. L929 cells and MCF-10A cells were chosen to investigate the biocompatibility of the ZC-DOX@GEL hydrogel. Cytotoxicity toward these cells was evaluated using the CCK-8 assay (Fig. 3B,C). The results demonstrated that hydrogels containing ZC-DOX NPs exhibited relatively favorable cell viability. To assess the therapeutic effect of hydrogels in vitro, 4T1 cells were co-cultured with the hydrogels. Compared to the Control and GEL groups, the remarkable combined treatment effect of ZC-DOX@GEL was visually evident in the live/dead staining images. These images revealed a more intense red fluorescence, indicating a higher number of dead cells (Fig. 3D,E). Besides, we used Transwell experiments to analyze the effects of NPs containing hydrogels on the vertical migration of cancer cells. Crystal violet staining images showed that less migrated 4T1 cells were observed in the ZC-DOX@GEL group 24 h than in the control group (Fig. 3F,G).

### The ROS scavenging behaviors and anti-inflammation effects of injectable hydrogels

**Figure 4:**
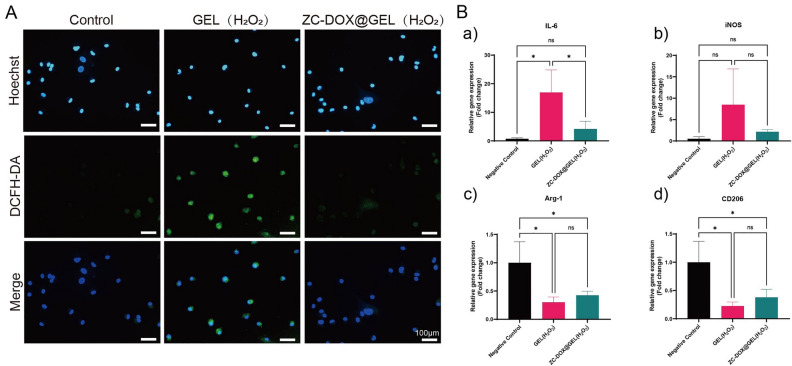
In vitro antioxidant and anti-inflammatory tests. (**A**) Intracellular ROS scavenging by ZC-DOX@GEL hydrogels in a H_2_O_2_-treated rat BMDMs cells. (**B**) Expression of inflammatory mediators (**a**) IL-6, (**b**) iNOS, (**c**) Arg-1 and (**d**) CD206 in LPS-treated cells.

The anti-oxidant and anti-inflammatory properties of ZC@GEL hydrogels were investigated at the cellular level. Rat BMDMs were stimulated with H_2_O_2_ or LPS (lipopolysaccharide), the latter being a potent proinflammatory polysaccharide derived from the Gram-negative bacterial cell wall. The DCFH-DA probe was utilized to detect intracellular reactive oxygen species (ROS). As shown in the fluorescence images (Fig. 4A), ROS generation in H2O2-treated cells exhibited a sharp increase. However, in the presence of ZC@GEL hydrogels, a negligible green fluorescence was observed in the BMDM cells, indicating a significant reduction in cellular ROS levels.

To gain further insight into the anti-inflammatory properties of ZC@GEL hydrogel, quantitative analysis was conducted on the changes in IL-6, iNOS, Arg-1, and CD206 gene expression (Fig. 4B). The treatment with ZC@GEL hydrogel efficiently suppressed the expression of pro-inflammatory genes (IL-6 and iNOS), while preserving the expression of Arg-1 and CD206 (Fig. 4B).

### In vivo tumor recurrence prevention by ZC-DOX@GEL

**Figure 5:**
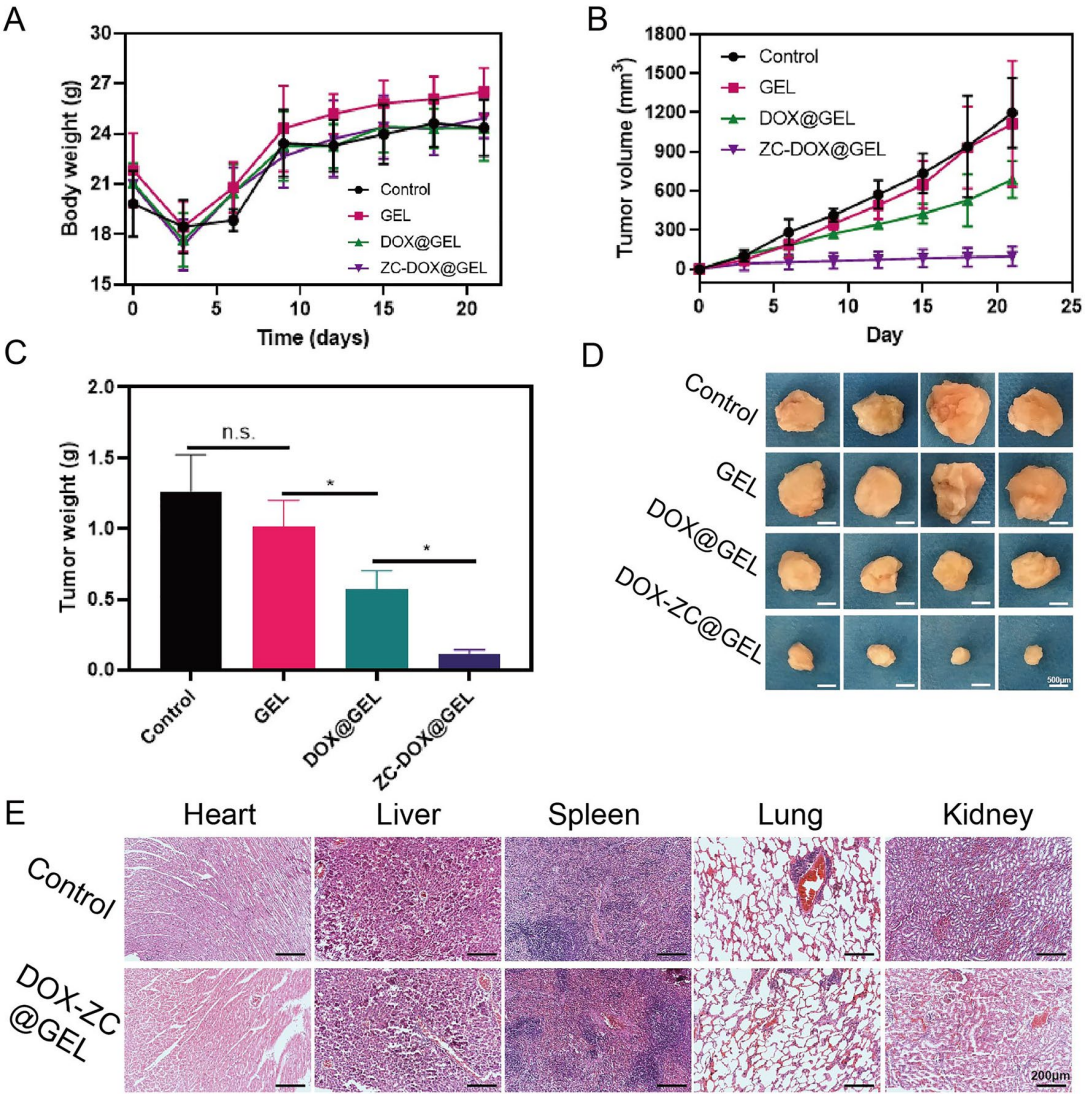
In vivo anticancer effect to 4T1 bearing balb/c mice (n = 4). (**A**) Body weight of 4T1 bearing balb/c mice as a function of time after different treatments. (**B**) H&E staining of major organs of balb/c mice treated with PBS or DZCG hydrogel, respectively. (**C**) Tumor volume of 4T1 bearing balb/c mice as a function of time after different treatments. (**D**) Tumor weights of 4T1 tumor-bearing mice with various treatments. (**E**) Photographs of the excised tumors.

Based on our in vitro experiments, we administered postoperative treatments of DC-DOX@GEL to Balb/c mice with breast tumors. Following surgery, all mice experienced temporary weight loss, noted on the third day (Fig. 5A), attributed to surgical trauma. Subsequently, the body weights of mice in various treatment groups increased, indicating overall health improvement. Throughout the treatment duration, variations in tumor volume among the groups were observed (Fig. 5B). The PBS and GelMA hydrogel groups showed moderate tumor growth over the testing period. In contrast, both the DOX@GEL group and the ZC-DOX@GEL group exhibited slight tumor growth, with the ZC-DOX@GEL group demonstrating a more pronounced therapeutic effect (Fig. 5C). After 21 days of observation and measurement, mice from all three groups were euthanized, and tissue samples were collected. Photographs of excised tumors are depicted in Fig. 5D, corroborating in vitro assay results and confirming that ZC-DOX NPs can partially inhibit tumor growth with reduced toxicity and enhanced efficacy compared to DOX alone. The in vivo safety of DC-DOX@GEL was further assessed through H&E staining (Fig. 5E) of major organs 24 h after postoperative treatments. No discernible damage was observed in vital organs (heart, liver, spleen, lungs, and kidneys), indicating minimal toxicity associated with DC-DOX@GEL treatment.

### Wound healing effects of ZC-DOX@GEL

**Figure 6:**
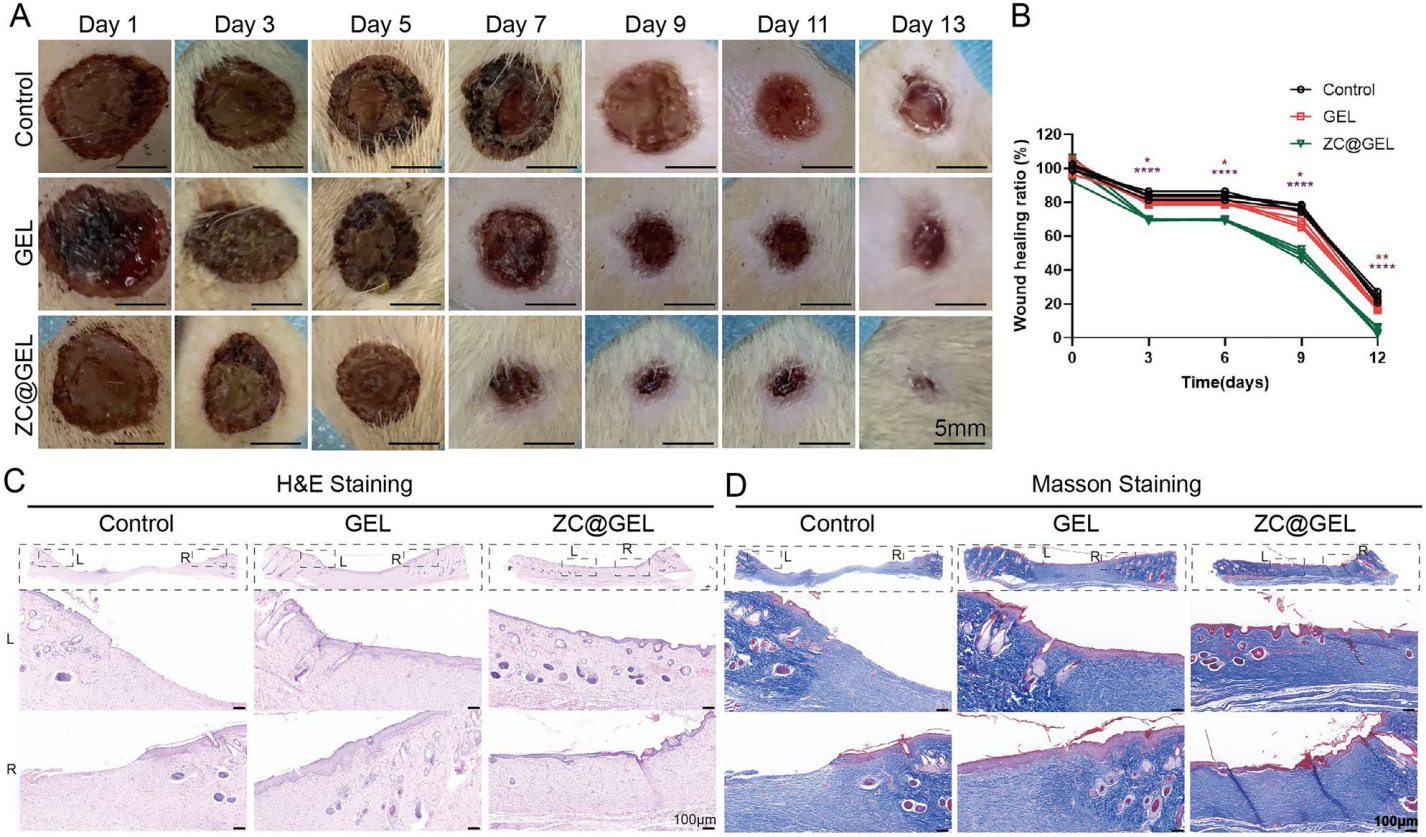
Wound healing ability. (**A**) Photography of representative wounds at different time after different treatments (scale bar: 5 mm). (**B**) Quantitative evaluation of wound healing ratio as a function of time after treatment. H&E (**C**) and Masson (**D**) staining of healing wounds from rat 13 days received different treatments, respectively (scale bar: 100 μm.

A full-thickness excisional skin model in rats was established to simulate the wound healing process post-surgery in vivo. The mice were divided into three groups: Control (treated with PBS only), GEL (treated with GelMA hydrogel only), and ZC@GEL (treated with ZIF-8@CeO_2_-loaded GelMA hydrogel). On predetermined days, the wounded areas were photographed and the changes in the wounds were recorded. The images displayed varying degrees of wound healing in the three groups (Fig. 6A and B). Statistically, the hydrogel-treated wounds exhibited significantly faster healing compared to the control group over time. Specifically, the ZC@GelMA group demonstrated the most robust healing results, with wound closure area reaching nearly 90% after 12 days. The wound closure for the control and GEL groups achieved 76.3% and 82.38%, respectively. These blended hydrogels exhibited outstanding wound healing behavior.

To further investigate the wound closure performance of the hydrogels, H&E staining was employed. Histological analysis of the wound sites revealed varying degrees of healing in these groups after treatment for 13 days (Fig. 6C,D). Compared with the other groups, the ZC@GelMA group showed superior repair effects, with epithelial and connective tissues exhibiting a more regular structure and the presence of hair follicles on the wound sites. At higher magnification, the overall organization of the skin appeared basically normal in the ZC@GelMA group, indicating a clear structure of the stratum corneum. Additionally, abundant collagen deposition in the GelMA and DC@GelMA groups indicated satisfactory skin repair. These results collectively suggest that the mitigation of inflammation and accelerated wound healing can be achieved with DC@GEL treatment.

## Discussion

Preventing tumor recurrence and promoting wound healing are paramount concerns following surgical resection. Injectable hydrogels hold great promise as matrices and multifunctional platforms for the immediate elimination of residual tumor cells and as long-term aids in wound healing. Additionally, various bioactive hydrogels have been designed to enhance tissue regeneration by providing physical or chemical cues^26^. Hydrogels endowed with the capacity to scavenge reactive oxygen species (ROS) play a crucial role in tissue regeneration. They shield cells from oxidative stress and facilitate the polarization of macrophage cells towards the M2 phenotype^27–29^. Despite considerable efforts to enhance anticancer efficacy through multimodal therapies and to foster wound healing via anti-inflammatory strategies, there remains a need for an adaptable matrix that supports tissues and ensures continuous ROS scavenging for improved tumor postoperative treatment.

In this study, we engineered ZIF-8@CeO_2_ nanoparticles loaded with DOX. The presence of both the ZIF-8 shell and CeO_2_ components within the nanoparticles ensured that the integrated drug delivery system exhibited a higher affinity towards cells and could respond effectively to the acidic microenvironment of tumors. The pathway for DOX diffusion from ZC-DOX@GEL involved a drug-containing core and the hydrogel matrix. Prior to being released from the spaces between GelMA chains, DOX had to traverse the network composed of water-filled pores in the ZIF-8@CeO_2_ nanoparticle. This multi-faceted diffusion mechanism endowed ZC-DOX@GEL with a slower initial release, thereby mitigating the issue of burst release commonly observed in free drug-loaded hydrogels. Furthermore, it has been established that nanomedicines integrated into hydrogels extend the retention time of drugs in the vicinity of tumors^30,31^. Our release experiments demonstrated that ZC-DOX@GEL exhibited a gradual initial burst followed by sustained DOX release. This drug release profile aligns well with the requirements for effective tumor postoperative treatments.

Furthermore, zinc ions released from ZIF-8@CeO_2_ nanoparticle might play a crucial role in regulating cellular antioxidant defense mechanisms. They participate in various mechanisms within cells to maintain the balance of oxidation–reduction processes and intra microenvironment stability, thus aiding in the neutralization of harmful ROS^32,33^. Specifically, zinc ions interact with antioxidant enzymes such as superoxide dismutase and catalase, as well as metallothionein, enhancing their activity and stability^34^. These enzymes and molecules work to eliminate free radicals and other detrimental oxidative substances, thereby reducing cellular damage caused by oxidative stress. Additionally, zinc ions influence intracellular redox reactions, modulating the cellular oxidation–reduction state. They form stable complexes with thiols (sulfur-containing molecules) and proteins, thereby affecting the cellular redox balance^35^. By regulating redox reactions, zinc ions reduce the production of free radicals within cells, preventing cellular damage induced by oxidative stress. Therefore, zinc ions might play a pivotal role in cellular antioxidant defense by enhancing antioxidant enzyme activity, regulating redox reactions, and maintaining intracellular redox balance. Maintaining appropriate levels by controlled release of zinc ions from ZIF-8@CeO_2_ nanoparticle is essential for cellular antioxidant defense and would healing.

The rheological analysis (Fg. 2I and J) consistently demonstrated solid-like elastic behavior, a characteristic trait of hydrogels, as indicated by the storage modulus (G’) surpassing the loss modulus (G’’) across the frequency range^36,37^. The injectability of our fabricated hydrogels stems from their specific shear-thinning behavior. The incorporation of ZC-DOX NPs in the hydrogel matrix provides a reservoir for chemotherapeutic drugs, such as DOX, enabling efficient tumor elimination (Fig. 3D). Moreover, ROS, generated in wounds due to bacterial infection or by normal cells at the wound site, can accumulate excessively, triggering an inflammatory response that impedes tissue repair and damages healthy cells^38,39^. The CeO_2_ component exhibits CAT-like and SOD-like catalytic activities, along with the ability to scavenge hydroxyl radicals in the hydrogel system, ensuring sustained ROS scavenging (Fig. 4A and B)^40,41^. Excessive levels of ROS can lead to elevated levels of inflammation through directly modifying signaling proteins or by inducing oxidative stress and then lead to the maturation and secretion of pro-inflammatory cytokines such as interleukin-1β (IL-1β) and interleukin-18 (IL-18)^42,43^. Additionally, excessive ROS can induce the expression of pro-inflammatory genes by activating transcription factors such as NF-κB and activator protein-1 (AP-1)^44,45^. These transcription factors bind to the promoters of pro-inflammatory genes and stimulate their transcription, leading to the production of cytokines, chemokines, and adhesion molecules that recruit immune cells to the site of inflammation^46,47^.

The relationship between inflammation and wound repair is a complex and dynamic process that involves various cellular and molecular interactions^38,48,49^. When tissue damage occurs, inflammatory cells such as neutrophils and macrophages are recruited to the site of injury, where they release cytokines and chemokines, promoting inflammation and attracting other immune cells to clear debris and pathogens from the wound area, thereby initiating a crucial initial inflammatory response^50^. Inflammatory cells release growth factors and cytokines that stimulate various aspects of the wound healing process. For example, transforming growth factor-beta (TGF-β) is released by macrophages and plays a central role in promoting the proliferation of fibroblasts and the synthesis of extracellular matrix proteins such as collagen^51^. Additionally, inflammatory cytokines such as interleukin-1 (IL-1) and tumor necrosis factor-alpha (TNF-α) stimulate the migration of keratinocytes and fibroblasts to the wound site^52^. However, failure to resolve inflammation can lead to chronic inflammation and impaired wound healing^53^. As the wound healing process progresses, anti-inflammatory signals are released to dampen the inflammatory response and promote tissue repair. Macrophages undergo a phenotypic switch from a pro-inflammatory (M1) phenotype to an anti-inflammatory (M2) phenotype, which promotes tissue remodeling and angiogenesis^54^. Additionally, inflammation also promotes angiogenesis, the formation of new blood vessels, which is essential for delivering oxygen and nutrients to the wound site^55^.

Both in vivo and in vitro experiments unequivocally confirmed the prevention of tumor recurrence and the acceleration of wound healing. Local chemotherapy administration demonstrated remarkable anticancer effects, leading to complete elimination of residual tumors. Furthermore, the injectable hydrogels induced a notably accelerated wound healing process compared to commercial wound dressings (Fig. 6). Hence, these multifunctional platforms, based on injectable hydrogels integrating multi-modal cancer therapies and ROS scavenging, present themselves as promising candidates for postoperative tumor treatments.

## Conclusion

In conclusion, we have successfully developed an inflammation-responsive injectable hydrogel system loaded with DOX, demonstrating potent localized tumor-suppressive and wound-healing effects. The notable antitumor efficacy of the composite hydrogels arises from the pH-sensitive release of DOX precisely at tumor sites. Concurrently, these composite hydrogels exhibit remarkable capabilities for efficient wound repair, achieved through the alleviation of wound-related inflammation and the creation of a conducive reparative microenvironment. Collectively, these findings offer valuable insights for the advancement of a postoperative adjuvant platform, effectively addressing clinical challenges associated with surgery, including wound healing post-resection and the prevention of tumor recurrence. This work not only introduces a valuable adjunct for postoperative therapy but also presents a straightforward methodology for synergistically harnessing diverse substances to achieve a myriad of biological applications.

### Supplementary Information

Supplementary Information.

## Data Availability

The data that support the findings of this study are available from the corresponding author upon reasonable request.

## References

[1] Wang X, Wu B, Zhang Y, Dou X, Zhao C, Feng C (2022). Polydopamine-doped supramolecular chiral hydrogels for postoperative tumor recurrence inhibition and simultaneously enhanced wound repair. Acta Biomater..

[2] Davis LE, Shalin SC, Tackett AJ (2019). Current state of melanoma diagnosis and treatment. Cancer Biol. Ther..

[3] Hiller JG, Perry NJ, Poulogiannis G, Riedel B, Sloan EK (2018). Perioperative events influence cancer recurrence risk after surgery. Nat. Rev. Clin. Oncol..

[4] Fearon K, Arends J, Baracos V (2013). Understanding the mechanisms and treatment options in cancer cachexia. Nat. Rev. Clin. Oncol..

[5] Kouhbananinejad SM, Armin F, Dabiri S, Derakhshani A, Iranpour M, Farsinejad A (2018). Application and assessment of allogeneic fibroblasts for cell therapy. Iran. J. Pathol..

[6] Ceelen W, Pattyn P, Mareel M (2014). Surgery, wound healing, and metastasis: recent insights and clinical implications. Crit. Rev. Oncol. Hematol..

[7] Chen B, Yang Z, Zhu Y, Xia Y (2014). Zeolitic imidazolate framework materials: Recent progress in synthesis and applications. J. Mater. Chem. A.

[8] Stock N, Biswas S (2012). Synthesis of metal-organic frameworks (MOFs): Routes to various MOF topologies, morphologies, and composites. Chem. Rev..

[9] Furukawa H, Cordova KE, O’Keeffe M, Yaghi OM (2013). The chemistry and applications of metal-organic frameworks. Science.

[10] Qiu J, Tomeh MA, Jin Y, Zhang B, Zhao X (2023). Microfluidic formulation of anticancer peptide loaded ZIF-8 nanoparticles for the treatment of breast cancer. J. Colloid Interface Sci..

[11] Hoseinpour V, Shariatinia Z (2021). Applications of zeolitic imidazolate framework-8 (ZIF-8) in bone tissue engineering: A review. Tissue Cell.

[12] Hao J, Yan Q, Li Z, Liu X, Peng J, Zhang T, Li J, Li D, He D, Zhou D (2022). Multifunctional miR181a nanoparticles promote highly efficient radiotherapy for rectal cancer. Mater. Today Adv..

[13] Wang G, Zhang J, He X, Zhang Z, Zhao Y (2017). Ceria nanoparticles as enzyme mimetics. Chin. J. Chem..

[14] Wang S, Yao Z, Zhang X, Li J, Huang C, Ouyang Y, Qian Y, Fan C (2022). Energy-supporting enzyme-mimic nanoscaffold facilitates tendon regeneration based on a mitochondrial protection and microenvironment remodeling strategy. Adv. Sci..

[15] Hua J, Wu P, Gan L, Zhang Z, He J, Zhong L, Zhao Y, Huang Y (2021). Current strategies for tumor photodynamic therapy combined with immunotherapy. Front. Oncol..

[16] Xu X, Mao H, Wu Y, Liu S, Liu J, Li Q, Yang M, Zhu J, Zou S, Du F (2022). Fabrication of methylene blue-loaded ovalbumin/polypyrrole nanoparticles for enhanced phototherapy-triggered antitumour immune activation. J. Nanobiotechnol..

[17] Sun C, Wang Z, Yue L, Huang Q, Cheng Q, Wang R (2020). Supramolecular induction of mitochondrial aggregation and fusion. J. Am. Chem. Soc..

[18] Tong Z, Guo Q, Xu G, Gao Y, Yang H, Ding Y, Wang W, Mao Z (2022). Supramolecular hydrogel-loaded Prussian blue nanoparticles with photothermal and ROS scavenging ability for tumor postoperative treatments. Composit. Part B: Eng..

[19] Dong K, Xu C, Ren J, Qu X (2022). Chiral nanozymes for enantioselective biological catalysis. Angew. Chem. Int. Ed..

[20] Norouzi M, Nazari B, Miller DW (2016). Injectable hydrogel-based drug delivery systems for local cancer therapy. Drug Discov. Today.

[21] Zhang A, Liu Y, Qin D, Sun M, Wang T, Chen X (2020). Research status of self-healing hydrogel for wound management: A review. Int. J. Biol. Macromol..

[22] Xu Q, Sigen A, Gao Y, Guo L, Creagh-Flynn J, Zhou D, Greiser U, Dong Y, Wang F, Tai H (2018). A hybrid injectable hydrogel from hyperbranched PEG macromer as a stem cell delivery and retention platform for diabetic wound healing. Acta Biomater..

[23] Tsai L-H, Young T-H, Yen C-H, Yao W-C, Chang C-H (2023). Intratumoral thermo-chemotherapeutic alginate hydrogel containing doxorubicin loaded PLGA nanoparticle and heating agent. Int. J. Biol. Macromol..

[24] Kim CK, Kim T, Choi IY, Soh M, Kim D, Kim YJ, Jang H, Yang HS, Kim JY, Park HK (2012). Ceria nanoparticles that can protect against ischemic stroke. Angew. Chem. Int. Ed..

[25] Yang J, Zhang X, Lu B, Mei J, Xu L, Zhang X, Su Z, Xu W, Fang S, Zhu C (2023). Inflammation-responsive hydrogel spray for synergistic prevention of traumatic heterotopic ossification via dual-homeostatic modulation strategy. Adv. Sci..

[26] He W, Reaume M, Hennenfent M, Lee BP, Rajachar R (2020). Biomimetic hydrogels with spatial-and temporal-controlled chemical cues for tissue engineering. Biomater. Sci..

[27] Zhou J, Liu W, Zhao X, Xian Y, Wu W, Zhang X, Zhao N, Xu FJ, Wang C (2021). Natural melanin/alginate hydrogels achieve cardiac repair through ROS scavenging and macrophage polarization. Adv. Sci..

[28] Huang C, Dong L, Zhao B, Lu Y, Huang S, Yuan Z, Luo G, Xu Y, Qian W (2022). Anti-inflammatory hydrogel dressings and skin wound healing. Clin. Transl. Med..

[29] Zhang S, Liu Y, Zhang X, Zhu D, Qi X, Cao X, Fang Y, Che Y, Han Z-C, He Z-X (2018). Prostaglandin E2 hydrogel improves cutaneous wound healing via M2 macrophages polarization. Theranostics.

[30] Brachi G, Ruiz-Ramirez J, Dogra P, Wang Z, Cristini V, Ciardelli G, Rostomily RC, Ferrari M, Mikheev AM, Blanco E (2020). Intratumoral injection of hydrogel-embedded nanoparticles enhances retention in glioblastoma. Nanoscale.

[31] Park SH, Kim DY, Panta P, Heo JY, Lee HY, Kim JH, Min BH, Kim MS (2017). An intratumoral injectable, electrostatic, cross-linkable curcumin depot and synergistic enhancement of anticancer activity. NPG Asia Mater..

[32] Hübner C, Haase H (2021). Interactions of zinc-and redox-signaling pathways. Redox Biol..

[33] Guo H, Peng X, Dong X, Li J, Cheng C, Wei Q (2023). Promoting stem cell mechanosensing and osteogenesis by hybrid soft fibers. ACS Appl. Mater. Interfaces.

[34] Yang F, Smith MJ, Griffiths A, Morrell A, Chapple SJ, Siow RC, Stewart T, Maret W, Mann GE (2023). Vascular protection afforded by zinc supplementation in human coronary artery smooth muscle cells mediated by NRF2 signaling under hypoxia/reoxygenation. Redox Biol..

[35] Franco C, Canzoniero LMT (2023). Zinc homeostasis and redox alterations in obesity. Front. Endocrinol..

[36] Zhou D, Li S, Pei M, Yang H, Gu S, Tao Y, Ye D, Zhou Y, Xu W, Xiao P (2020). Dopamine-modified hyaluronic acid hydrogel adhesives with fast-forming and high tissue adhesion. ACS Appl. Mater. Interfaces.

[37] Pugliese R, Gelain F (2020). Characterization of elastic, thermo-responsive, self-healable supramolecular hydrogel made of self-assembly peptides and guar gum. Mater. Des..

[38] Zhang W, Dai X, Jin X, Huang M, Shan J, Chen X, Qian H, Chen Z, Wang X (2023). Promotion of wound healing by a thermosensitive and sprayable hydrogel with nanozyme activity and anti-inflammatory properties. Smart Mater. Med..

[39] Zhou J, Fang C, Rong C, Luo T, Liu J, Zhang K (2023). Reactive oxygen species-sensitive materials: A promising strategy for regulating inflammation and favoring tissue regeneration. Smart Mater. Med..

[40] Zhao L, Peng B, Hernandez-Viezcas JA, Rico C, Sun Y, Peralta-Videa JR, Tang X, Niu G, Jin L, Varela-Ramirez A (2012). Stress response and tolerance of Zea mays to CeO_2_ nanoparticles: Cross talk among H_2_O_2_, heat shock protein, and lipid peroxidation. ACS Nano.

[41] Sun Y, Liu X, Wang L, Xu L, Liu K, Xu L, Shi F, Zhang Y, Gu N, Xiong F (2022). High-performance SOD mimetic enzyme Au@ Ce for arresting cell cycle and proliferation of acute myeloid leukemia. Bioact. Mater..

[42] Bai Y, Li Z, Liu W, Gao D, Zhang P, Liu M (2019). Effects of IL-1β and IL-18 induced by NLRP3 inflammasome activation on myocardial reperfusion injury after PCI. Eur. Rev. Med. Pharmacol. Sci..

[43] Wang XJ, Ni XQ, Zhao S, Zhao RZ, Wang XH, Xia SJ, Sun XW, Zhuo J (2022). ROS–NLRP3 signaling pathway induces sterile inflammation after thulium laser resection of the prostate. J. Cell. Physiol..

[44] Lin B-R, Yu C-J, Chen W-C, Lee H-S, Chang H-M, Lee Y-C, Chien C-T, Chen C-F (2009). Green tea extract supplement reduces D-galactosamine-induced acute liver injury by inhibition of apoptotic and proinflammatory signaling. J. Biomed. Sci..

[45] Shi Z, Li X, Chen J, Dai Z, Zhu Y, Wu T, Liu Q, Qin H, Zhang Y, Chen H (2024). Enzyme-like biomimetic oral-agent enabling modulating gut microbiota and restoring redox homeostasis to treat inflammatory bowel disease. Bioact. Mater..

[46] Blaser H, Dostert C, Mak TW, Brenner D (2016). TNF and ROS crosstalk in inflammation. Trends Cell Biol..

[47] Chang M, Nguyen TT (2021). Strategy for treatment of infected diabetic foot ulcers. Acc. Chem. Res..

[48] Cai Y, Chen K, Liu C, Qu X (2023). Harnessing strategies for enhancing diabetic wound healing from the perspective of spatial inflammation patterns. Bioact. Mater..

[49] Jeschke MG, Wood FM, Middelkoop E, Bayat A, Teot L, Ogawa R, Gauglitz GG (2023). Scars. Nat. Rev. Dis. Prim..

[50] Sreejit G, Johnson J, Jaggers RM, Dahdah A, Murphy AJ, Hanssen NM, Nagareddy PR (2022). Neutrophils in cardiovascular disease: Warmongers, peacemakers, or both?. Cardiovasc. Res..

[51] Vignola A, Chanez P, Chiappara G, Merendino A, Zinnanti E, Bousquet J, Bellia V, Bonsignore G (1996). Release of transforming growth factor-beta (TGF-β) and fibronectin by alveolar macrophages in airway diseases. Clin. Exp. Immunol..

[52] Fedyk ER, Jones D, Critchley HO, Phipps RP, Blieden TM, Springer TA (2001). Expression of stromal-derived factor-1 is decreased by IL-1 and TNF and in dermal wound healing. J. Immunol..

[53] Eming SA, Krieg T, Davidson JM (2007). Inflammation in wound repair: molecular and cellular mechanisms. J. Investig. Dermatol..

[54] Italiani P, Boraschi D (2014). From monocytes to M1/M2 macrophages: Phenotypical vs. functional differentiation. Front. Immunol..

[55] Szade A, Grochot-Przeczek A, Florczyk U, Jozkowicz A, Dulak J (2015). Cellular and molecular mechanisms of inflammation-induced angiogenesis. IUBMB Life.

