# Supplementary material for: Injectable hydrogel with doxorubicin-loaded ZIF-8 nanoparticles for tumor postoperative treatments and wound repair
Source: Sci Rep. 2024 May 1;14:9983. doi: 10.1038/s41598-024-57664-0 (PMC11063161; doi:10.1038/s41598-024-57664-0)
Supplement: Supplementary file 1 — Supplementary Information. [file 41598_2024_57664_MOESM1_ESM.docx]

Supplementary Information for

**Injectable hydrogel** **with doxorubicin-loaded ZIF-8 nanoparticles for tumor postoperative treatments and wound repair**

## Supplementary Materials and Methods

**1. Applied Chemicals.**

2-Methylimidazole (2-MeIM) and zinc acetate (Zn (Ac)_2_) were purchased from Aladdin Reagent (Shanghai, China). All chemicals had purity ≥ 99 % and all organic solvents had purity ≥ 99.7 %. Cerium (III) acetate hydrate (99.9%), Oleylamine (80−90%) were purchased from Sigma-Aldrich (USA).

**2. Other applied materials**

The Dulbecco’s Modified Eagle Medium (DMEM), phosphate buffered saline (PBS), fetal bovine serum (FBS), Trypsin were all sourced from Procell (Wuhan, China). Propidium iodide (PI), Calcein AM, Hoechst 33342, and 2′,7′- Dichlorodihydrofluorescein diacetate (DCFH-DA) were purchased from Beyotime (Shanghai, China).

**3. Cell culture**

The Mouse macrophages (RAW264.7) and 4T1 cells and were acquired from Procell Co., Ltd (Wuhan, China). Bone marrow-derived macrophage (BMDM) isolation and induction were as follows. Briefly, femurs and tibias were obtained aseptically from SD rats (6 weeks old). After rinsing the cavity with a syringe, the bone marrow was filtered through a 100 µm filter and subsequently processed by erythrocyte lysis. Then, the cells were cultured in DMEM (Gibco) supplemented with 10% inactivated FBS (Gibco), 100 U/ml penicillin– streptomycin (Gibco), and 40 ng/ml recombinant macrophage-colony stimulating factor (M-CSF, Peprotech) for a 7-day induction to obtain mature BMDMs.

**4. Synthesis of gelatin methacryloyl (GelMA)**

GelMA was synthesized, from porcine skin gelatin type A, according to the general method first adopted by Van Den Bulcke et al[1]. In brief, gelatin was mixed at 10% (w/v) into dPBS at 60 °C and stirred until fully dissolved. Then, 0.6 g of MA/1 g of gelatin was added dropwise to the gelatin solution, at 50 °C and a rate of 0.5 mL min−1 under stirring and allowed to react for 1 h. After 1 h, the reaction was stopped following 5× dilution with warm (40 °C) dPBS. To remove salts and unreacted MA, the mixture was dialyzed for 5 days at 40 °C against distilled water using 12–14 kDa cutoff dialysis tubing, in the dark. The solution was finally freeze-dried, generating a porous white foam that was stored at -20 °C until further use.

**Table S1**. Primer sequences used for the in vivo RT–qPCR analysis of inflammatory genes.

| Gene | Forward Primers | Reverse Primers |
| --- | --- | --- |
| iNOS | ACATCGACCCGTCCACAGTAT | CAGAGGGGTAGGCTTTGTCTC |
| IL-6 | TCTCTGGGAAATCGTGGAAATGA | GGTACTCCAGAAGACCAGAGGA |
| CD206 | GATGGATGGGAGCAAAGTAGAT | GTCATTCCAGAACCCTGAATTTG |
| Arg-1 | ACCTGGCCTTTGTTGATGTCCCTA | AGAGATGCTTCCAACTGCCAGACT |
| GAPDH | TTGTCTCCTGCGACTTCAAC | TTACTCCTTGGAGGCCATGT |

[1] A.I. Van Den Bulcke, B. Bogdanov, N. De Rooze, E.H. Schacht, M. Cornelissen, H. Berghmans, Structural and rheological properties of methacrylamide modified gelatin hydrogels, Biomacromolecules 1(1) (2000) 31-38.
